# Environmental fate of nanopesticides: durability, sorption and photodegradation of nanoformulated clothianidin†
†Electronic supplementary information (ESI) available. See DOI: 10.1039/c8en00038g


**DOI:** 10.1039/c8en00038g

**Published:** 2018-02-22

**Authors:** Melanie Kah, Helene Walch, Thilo Hofmann

**Affiliations:** a Department of Environmental Geosciences and Environmental Science Research Network , University of Vienna , Althanstrasse 14, 5 UZA2 , 1090 Vienna , Austria . Email: Melanie.kah@univie.ac.at ; Email: thilo.hofmann@univie.ac.at; b Commonwealth Scientific and Industrial Research Organisation (CSIRO) , Waite Campus, Locked Bag No 2 , Glen Osmond , SA 5064 , Australia

## Abstract

A lot of research efforts are currently dedicated to the development of nano-enabled agrochemicals. Knowledge about their environmental behaviour is however scarce, which impedes the assessment of the new risk and benefits relative to currently used agrochemicals. With the aim to advance our understanding of the fate of nanopesticides in the environment and support the development of robust exposure assessment procedures, the main objectives of the study were to (i) investigate the extent to which three nanoformulations can affect the photodegradation and sorption of the insecticide clothianidin, and (ii) evaluate various approaches to estimate durability, a key parameter for the exposure assessment of nanopesticides. The nanoformulations increased the photodegradation half-life in water by a maximum of 21% relative to the conventional formulation. Sorption to soil was investigated by two methods and over time, and results show that sorption was increased by up to 51% and 10%, relative to unformulated clothianidin and the commercial formulation, respectively. Our results generally indicate that nanoformulations may have a greater impact on the fate of pesticide active ingredients than commercial formulations. It is important to note however that differences in fate parameters were generally very moderate, including in realistic worst-case conditions (high pesticide concentration and ionic strength). Our results collectively suggest that clothianidin was rapidly released from the nanocarrier systems and that the durability of the three nanoformulations would be short in water as well as in soil environments (including under realistic soil to solution ratio). The durability of nanoformulations after their application in the environment is an essential parameter that needs to be characterised for the development as well as for the evaluation of nano-enabled agrochemicals. This study illustrates how performances of nano-enabled products can be critically benchmarked against existing products to support an objective assessment of new environmental risks and benefits. In this context, the fate of the nanocarrier system is of great interest and should be the topic of further research.

## Abstract 2

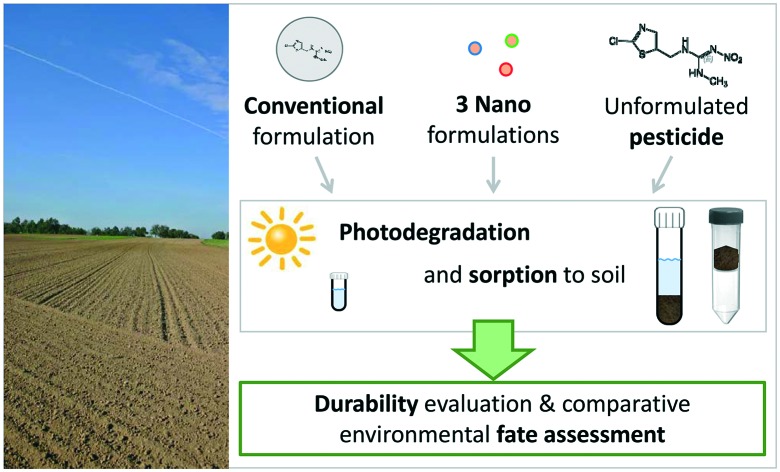
A lot of research efforts are currently dedicated to the development of nano-enabled agrochemicals. It is thus urgent to develop suitable strategies for their ecological assessment.

##

Environmental significance

Nanotechnology has the potential to support the development of more environment friendly agrochemicals and reduce the impact that agriculture has on the environment and human health. Very little is known about the environmental behaviour of nano-enabled agrochemicals, making the risks and benefits of the novel products difficult to assess relative to conventional agrochemicals. Focussing on photodegradation and sorption behaviour, we show how protocols in place within the pesticide assessment framework can be used to estimate the durability of nanopesticides in the environment. Results obtained under realistic worst-case scenarios for nanopesticides and non-nano counterparts can help regulators take informed decisions without having to systematically perform a comprehensive new nano-specific assessment.

## Introduction

Applications of nanotechnology in agriculture are currently receiving a lot of attention, and novel nano-enabled agrochemicals are now being evaluated for market authorisation.^1^,^2^ Nanotechnology is often presented as having the potential to reduce the impact that modern agriculture has on human and environmental health, but the novel agrochemicals are also associated with fears related to the potential unwanted environmental impact through increased exposure and toxicity to non-target organisms.^1^ Pesticides are particularly sensitive products from an environmental and human health perspective as they are designed to provoke a reaction on biological systems, often including lethal effects on the target organism. It is thus essential that the new risks and benefits associated with the use of nanopesticides are identified, adequately evaluated and compared with existing products.

The ecological risk assessment of nanopesticides is likely to differ from that of conventional pesticides^3^ and new parameters are needed to allow an adequate evaluation of the new products. The majority of products currently in development consists in nanocarrier systems loaded with a registered active ingredient already in use (AI, *e.g.* a molecule with insecticidal or herbicidal properties).^4^,^5^ For this type of products, a priority for exposure assessment is to establish the durability of the AI–nanocarrier complex upon application in the field.^3^,^5^ If the durability of the nanoformulation is very short, exposure is likely to be similar to that of conventional pesticide formulations, whose ingredients are generally assumed to dissociate and behave independently upon application in the field. If the nanocarrier–AI complex persists in the environment, a more complex assessment of exposure also considering the nanocarrier properties may be required.^2^,^5^

There are currently no standard protocols to measure the durability of the AI–nanocarrier complex. One approach consists in measuring the release rate of the AI from the nanocarrier. Release experiments have been presented in literature as part of the characterisation of nano-agrochemicals, but they were often carried out at unrealistically high concentrations and in deionised water. Results are thus unlikely to be representative of the pH, ionic strength and dilution factor that would occur when users dilute the pesticide concentrate in locally supplied water in order to prepare an application tank (which may contain several pesticides as well as fertilisers). Characterising the release rate of the AI from the nanocarrier in soil is also necessary to estimate the durability of nanopesticides after their application in the field. Data on release in soil are scarce mainly due to the challenges associated with direct measurements at realistic soil to solution ratios. Kah *et al.*^6^ have recently proposed to estimate the durability of nanopesticides in soil indirectly, *via* the impact that nanoformulations have on other environmental kinetic processes. The approach was successfully applied to the process of AI degradation in soil and allowed the determination of release half-lives for a series of nanoformulations loaded with the insecticide bifenthrin.^6^ The same approach could potentially be applied to other fate processes, but it has not been tested yet.

With the aim to advance our understanding of the fate of nanopesticides in the environment and to support the development of robust exposure assessment procedures, the main objectives of the study were to (i) investigate the extent to which nanoformulations can affect the photodegradation and sorption of a pesticide AI, and (ii) evaluate the possibility to use those fate processes to estimate the durability of the formulations.

We considered three polymer-based nanoformulations of clothianidin, a neonicotinoid systemic insecticide. Neonicotinoids are chemically similar to nicotine and act on the central nervous system of insects.^7^ The use of neonicotinoids is generally considered to pose relatively low risks to mammals when compared to other insecticides, but they have been recently a topic of great controversy due to their alleged impact on pollinators, and bees in particular. Their authorisation is currently under controversial discussion in the European Union^8^ as well as in the United States.^9^ Photodegradation and sorption experiments including the three nanoformulations, a conventional formulation and the pure AI clothianidin were carried out across a wide range of concentration representing realistic application scenarios as well as conditions under which the association of clothianidin and nanocarrier are likely to be enhanced (high concentration and/or presence of fertilisers potentially provoking salting out effects).

## Materials and methods

### Soils

Two standard surface soils were sampled by LUFA (Speyer, Germany) according to ISO standards and good laboratory practices in March 2013. The soils were air-dried, sieved at 2 mm and analysed by LUFA Speyer. The Sand (2.8% clay, 87.0% sand, pH = 5.1 and 0.7% organic carbon) was uncultivated, whereas the Loam (25.9% clay, 33.6% sand, pH = 7.2 and 2.3% organic carbon) was sampled from a meadow with apple trees. Detailed soil properties are presented in Table S1.† No pesticides or fertilisers were applied to the soils for a period of minimum four years before sampling.

### Chemicals

The analytical-grade Pestanal® standard of clothianidin ((*E*)-1-(2-chloro-5-thiazolylmethyl)-3methyl-2-nitroguanidine) was bought from Sigma Aldrich (99.9% pure). Clothianidin has a solubility in water of 340 mg L^–1^ (20 °C, pH 10).^7^ Analytical-grade acetonitrile was purchased from VWR (Germany) and all aqueous solutions were prepared with ultrapure water (Millipore, Elix5-Milli-Q Gradient). PowerPhos liquid fertiliser (NPK 10-34-0) was purchased from Hechenbichler (Austria) and contained 138 g L^–1^ NH_4_, 469 g L^–1^ P_2_O_5_ and traces of Fe (0.1%). Three polymer-based nanoformulations containing approximately 20% by weight of clothianidin (NFA, NFB, NFC) were provided by Vive Crop Protection, Inc. (Canada). They were produced by bead milling and the nano carriers exhibited a primary particle size <100 nm (personal communication). The commercial formulation “Belay” a suspension concentrate was purchased from an agricultural retail in the United States (Raleigh, NC). The composition of each formulation is shown in Table S2.†

### Characterisation of the nanoformulations

Hydrodynamic diameters and *ζ*-potentials were determined on the basis of dynamic light scattering and electrophoretic mobility measurements using laser Doppler velocimetry (ZetaSizer Nano ZS, Malvern, U.K., operating at 633 nm). Backscattered light was observed at 173°, and the autocorrelation function was fitted using the cumulant method, assuming a refractive index of 1.5. The *ζ*-potential was calculated from the electrophoretic mobility using Smoluchowski's equation. Measurements were performed after 1 : 400 dilutions (by volume) of the nanoformulations either with deionised water, NaCl 190 mM or with 75 mM Ca(NO_3_)_2_.^10^ Measurements in the presence of fertiliser were also performed to represent the conditions occurring in the photodegradation and sorption tests (details about the protocols are provided in the ESI,† together with Table S3).

The release rate of clothianidin from the polymer nanocarriers was measured using syringe filters (0.2 μm pore size, nylon, 25 mm diameter, Yeti Syringe Filters, Merz Brothers GmbH). Measurements were carried out at concentrations below and above the solubility of clothianidin, and in the presence or absence of fertiliser background. Suspensions of the nanoformulations were prepared at 6 and 600 mg L^–1^ of clothianidin and filtered without delay (within 5 min of preparation). The first 10 mL of filtrates were discarded; the subsequent fractions were collected and directly measured by high performance liquid chromatography (HPLC, see details below). Tests with solutions of non-formulated clothianidin indicated that there were no losses during the filtration procedure.

The influence of the fertiliser on the release rate was investigated with NFC (the formulation most prone to aggregation, see section on Characterisation). Suspensions were prepared following two protocols: either by the addition (i) of pure NFC in the fertiliser solution or (ii) of diluted NFC in deionised water. Clothianidin, NH_4_ and P_2_O_5_ concentrations were approximately 80, 2000 and 6800 mg L^–1^, respectively. The total concentration of clothianidin in the nanoformulations was determined after extraction with acetonitrile.

Clothianidin was quantified by HPLC, with a ZORBAX Eclipse XDB-C18 column (4.6 × 150 mm, 5 μm pore size) maintained at 30 °C and with a flow rate of 1 mL min^–1^. The mobile phase consisted of a mixture of deionised water and acetonitrile (starting with 100% water, increasing to 100% acetonitrile in seven minutes). Clothianidin eluted after 5.5 min, and was quantified at a wavelength of 269 nm based on calibration curves consisting of nine standards (0.1–15 mg L^–1^ in acetonitrile, *R*^2^ > 0.999). The limits of detection and quantification were 0.02 and 0.08 mg L^–1^, respectively.

### Photodegradation experiments

Photodegradation rates of clothianidin in the different formulations were determined following the OECD guidelines^11^ with slight modifications, and using an Atlas Suntest CPS+ equipped with a xenon lamp and an optical daylight filter (coated quartz filter) cutting off UV light at 290 nm. The irradiance was set to 65 W m^–2^ in the 300–400 nm range, which corresponds to natural summer irradiance in Basel (Switzerland, 50° N latitude^12^). Clothianidin suspensions in transparent screw-cap HPLC vials were placed randomly in the ageing chamber and irradiated for up to 6 h. At six time intervals, samples were taken out of the chamber and immediately stored in the fridge. Controls wrapped up in aluminium-foil were also placed in the chamber in order to account for the possible effects of temperature on clothianidin degradation. At the end of the experiments, all samples were extracted with acetonitrile (1 : 1 by volume) and the amount of clothianidin remaining over time was quantified by HPLC (see section on Characterisation).

Photodegradation experiments were conducted to determine the effect of (i) clothianidin concentration (in the range 13.6–544 mg L^–1^), and (ii) the presence of fertiliser. For the latter, concentrations were 136, 18 400, 62 560 and 184 mg L^–1^ for clothianidin, NH_4_, P_2_O_5_ and Fe, respectively, representing a foliar spray application scenario (see Table S4† for details on how concentrations were calculated).

The curves representing the concentration of clothianidin decreasing over time were fitted with a first-order kinetic model to determine the rate of photodegradation (*k*, day^–1^) and DT_50_ (time required for 50% of the initial dose to be photodegraded). No degradation occurred in the dark controls, except for NFC at the highest concentration investigated (see Fig. S1†). Losses did not exceed 3% of the initial concentration over the duration of the experiment (6 h), but degradation was significant (*p* = 0.022) and probably due to hydrolysis^7^ promoted by the high pH (NFC 8.08 ± 0.02 at the highest concentration considered in the degradation test) and temperature (up to 70 °C in the chamber). Results for NFC were corrected on the basis of the degradation curve observed in the dark controls.

### Sorption experiments

Sorption coefficients were determined by (i) a classical batch equilibrium method^13^ and (ii) a centrifugation method, which allows measuring sorption at realistic soil to solution ratios.^14^,^15^ Another objective of the sorption experiments was to determine the influence of fertiliser on the effect of formulation. Batch experiments are normally carried out in 0.01 M CaCl_2_ background solution, which leads to the precipitation of CaPO_4_ in the presence of fertiliser solution. Experiments were thus carried out either in deionized water or in fertiliser background solutions. Concentrations of clothianidin, NH_4_, P_2_O_5_ and Fe were 13, 320, 1088 and 3.2 mg kg^–1^ dry soil, respectively, and were chosen based on a realistic in-furrow application scenario (details are available in Table S4†).

For the batch experiments, 50 mL centrifugation tubes (PTFE, in triplicates) were filled with soil suspensions (10 g of soil and 19 mL of either deionised water or fertiliser solution), and pre-equilibrated overnight in the dark on a side-to side shaker (125 rpm). Suspensions were then spiked with either 1 mL of clothianidin solution (130 mg clothianidin per L in deionised water) or 0.1 mL of formulation (1300 mg clothianidin per L in deionised water) + 0.9 mL deionised water. Triplicate blanks for each soil confirmed the absence of clothianidin background and triplicate controls for each formulation were used to determine the total concentration of clothianidin. The samples were equilibrated through side-to-side shaking at 125 rpm in the dark for 24 h and then centrifuged for 30 min at 4000*g* (based on preliminary tests following the OECD guidelines^13^). The pH values were measured before and after sorption, and concentrations of clothianidin in the supernatants were measured by HPLC (see section on Characterisation). Sorption coefficients (*K*_d_, L kg^–1^) were calculated by mass balance.

Sorption was also measured using a centrifugation method applied to soils incubated at 60% of their maximum water holding capacity (equivalent to a soil : solution ratio of approximately 4 : 1 for the Sand and 5 : 1 for the Loam). The equivalent of 120 g of dry soil was weighed into 250 mL glass bottles with screw caps (Schott Duran). The moisture content was adjusted close to the desired value by addition of either deionised water or fertiliser solution. After careful mixing, samples were pre-incubated for three days in the dark and at 4 °C. The soils were then spiked with either 12 mL of clothianidin solution (130 mg clothianidin per L) or 1.2 mL of formulation (1300 mg clothianidin per L) and adjusted to exactly 60% of their maximum water holding capacity by weighing. Samples were thoroughly mixed and incubated in the dark at 4 °C. The moisture content was maintained by regular addition of deionised water throughout the incubation period.

After one and seven days of incubation, 10 g samples were weighed into the filter inserts of centrifuge tubes (VectaSpin 20, 50 mL, Whatman International Ltd.). The original filter was replaced by a polycarbonate membrane with a pore size of 0.4 μm (Whatman) to retain the nanocarriers (based on size measurements, see section on Characterisation), and a glass microfiber filter with a pore size of 1.6 μm (Whatman GF/A, *Ø* 25 mm) to retain soil particles and avoid scaling of the membrane. Filters and membranes were pre-wetted with 0.1 mL of H_2_O, and after the addition of the soil samples, the tubes were centrifuged for 30 min at 1500*g*, which applies a pressure of 200 kPa corresponding to the limit between “mobile” and “immobile” water.^16^ The volume of soil solution collected was about 0.2 and 1.0 mL for the Loam and Sand, respectively. The total concentration of clothianidin at one and seven days was determined from triplicate soil samples extracted with acetonitrile (10 g and 20 mL, respectively). After one hour side-to-side shaking at 125 rpm in the dark, the soil was left to settle down for one hour, and the supernatant was analysed by HPLC (see section on Characterisation). Recovery tests indicated a mean extraction efficiency of 103% (with a range of 93–113%). Sorption coefficients (*K*_d_, L kg^–1^) were calculated by mass balance.

### Statistics

Statistical analyses, curve fits and graphs were produced using GraphPad Prism 6 (GraphPad Software Inc. 2016). The significance level was set to *α* = 0.05. In the graphs, significant differences are indicated by * and error bars represent standard deviations (*n* = 3).

## Results and discussion

### Characterisation of the nanoformulations

**Fig. 1:**
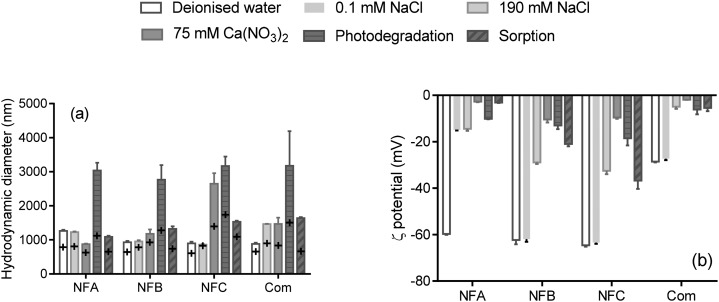
Hydrodynamic diameter (a) and *ζ*-potential (b) of three nanoformulations (NFA, NFB and NFC) and a commercial formulation (Com) of clothianidin, measured in a range of background solutions and in the conditions applied in the photodegradation and sorption tests (presence of fertiliser). The error bars represents the standard deviation for *n* = 3. On graph (a), the cross represents the first measurement upon mixing, and the bars are the average of three measurements at *t* > 8 min (see Table S3† for details).

Hydrodynamic diameters and *ζ*-potentials measured in a range of background solutions are shown in Fig. 1 (details are presented in Table S3†). Time resolved measurements (not shown) indicate that hydrodynamic diameters increased rapidly upon mixing the formulation with the background solutions, probably due to polymer swelling (size increased generally by less than two-fold, Fig. 1a). After about 10 minutes, the size of the particles in the three nanoformulations ranged from 900 nm up to 1264 nm in deionised water, which greatly exceeds the currently proposed threshold to define nanoparticles by the European Commission (100 nm (ref. 17)). Most nano-enabled agrochemicals exceed the size threshold of 100 nm whereas commercial formulations can unintentionally contain entities <100 nm,^4^,^18^,^19^ which illustrates the difficulties associated with defining nanopesticides solely based on a size criterion.

Hydrodynamic diameters were generally slightly greater in the presence of electrolytes than in deionised water, which can be explained by specific interactions with ions and/or conformational changes of the polymers or stabilizing agents impacting the diffusion behaviour of the nanocarriers. Aggregation (indicated by diameters at least twice larger than in deionised water) occurred for all formulations in the conditions representing the photodegradation test (relatively high concentration of fertiliser) as well as for NFC in the presence of Ca(NO_3_)_2_.

Changes in hydrodynamic diameter and *ζ*-potential due to the addition of salts can provide information on the main mode of colloidal stabilisation. Unlike NFB and NFC, the *ζ*-potential of NFA dropped from –60 mV in deionised water to –15 mV in 0.1 mM NaCl, and the presence of 75 mM Ca(NO_3_)_2_ further reduced the *ζ*-potential of NFA to –3 mV (Fig. 1). Size measurements indicate that aggregation did not occur for NFA in any of the background solutions, which suggests that steric stabilisation dominated in NFA. The *ζ*-potentials of both NFB and NFC dropped to about –10 mV (low electrostatic stability) in the presence of Ca(NO_3_)_2_, but only NFC aggregated. NFC was thus probably solely stabilised through electrostatic forces, whereas the stabilisation of NFB also involved steric forces.

The colloidal characteristics of the three nanoformulations are similar to a series of nanopesticides previously studied by Kah *et al.*^6^ and loaded with the insecticide bifenthrin, a very hydrophobic and persistent insecticide. Comparing the behaviour of the two series of nanoformulation will indicate the respective roles played by the nanocarrier properties and the AI properties on the nanopesticide behaviour (see the section on Sorption).

### Release of clothianidin from the nanocarriers

Measuring the release rate of clothianidin from the nanocarriers by filtration was unsuccessful. At concentrations below the solubility limit of clothianidin, there were no differences in concentration between the filtered and unfiltered samples. When the test was carried out at concentrations exceeding the solubility of clothianidin (up to 633, 656 and 1956 mg L^–1^ for NFA, NFB and NFC, respectively), concentration in the filtrates ranged 297–315 mg L^–1^, which corresponds to the water solubility of clothianidin.^7^ The results thus suggests that clothianidin was released from the nanocarriers very quickly upon dilution and that the association between clothianidin and the nanocarriers was mainly controlled by the solubility of the AI. The type of nanoformulations investigated was not designed for slow release purposes, but to increase tank-mix compatibility *e.g.* allowing the formation of homogeneous and sufficiently stable suspension with *e.g.* liquid fertilisers for combined application (personal communication). The durability of the AI–nanocarrier complex may be very limited in this case.

### Photodegradation

**Fig. 2:**
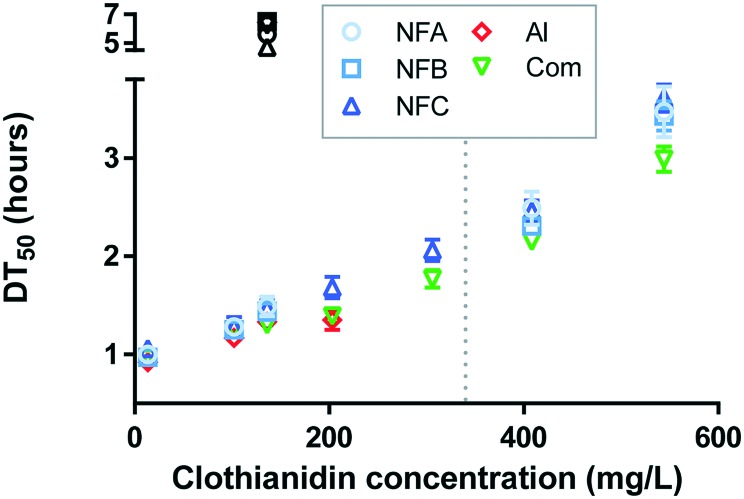
Photodegradation half-lives of clothianidin (DT_50_, hours) for the series of nanoformulations (NFA, NFB and NFC), the commercial formulation (Com) and pure clothianidin (AI). Tests were carried out across a wide range of concentrations either in water (coloured symbols) or in fertiliser background (black symbols). The dashed line indicates the water solubility of clothianidin (340 mg L^–1^ (ref. 7)).

Consistently with literature,^20^–^22^ photodegradation curves of clothianidin followed first-order kinetics (0.97 < *R*^2^ < 0.99), from which DT_50_ values were calculated (Fig. 2, all values are available in Table S5†).

In the water background, 0.92 < DT_50_ < 3.61 h, which corresponds to the range of values previously reported for pure clothianidin.^7^,^20^,^23^ As frequently observed,^24^–^27^ DT_50_ values increased as the concentration of clothianidin increased for all formulations and for the pure AI (about three-fold between the lowest and highest concentrations, *p* < 0.0001). The effect was not related to changes in pH (as pH increased with concentration in NFC and Com, but it decreased with concentration in NFA). The decrease in degradation with increasing concentration of clothianidin was thus probably due to shading effects.

Nanoformulations discussed so far in the literature could either slow down^28^–^31^ or catalyse^32^ the photodegradation of the associated AI. The DT_50_ of clothianidin generally followed the order AI < Com < NFB < NFA < NFC. Differences were generally not significant at the lowest concentration levels (all comparisons are graphically presented in Fig. S2†), but at concentrations close to and above the solubility limit, DT_50_ values were significantly higher for the three nanoformulations than for Com, suggesting that the nanoformulations studied can exert a protective effect against photodegradation.

Compared to the results obtained in water, photodegradation was significantly slower in the fertiliser background (average DT_50_ 5.9 h, black symbols in Fig. 2), probably due to light absorption (solutions with fertilisers were slightly coloured). Additional attenuation due to quenching or complexation with NH_4_^+^ or Fe is also possible (both were present at relatively high concentration in the fertiliser solution). In the fertiliser background, the degradation rate followed the order AI = Com = NFB < NFA < NFC, indicating that two out of the three NFs significantly accelerated the photodegradation of clothianidin in the presence of fertiliser (Fig. S2†). The mechanisms of clothianidin photodegradation have been studied^20^ and mainly consist in radical denitration. The influence of ammonium, phosphate and iron has not been investigated up to now. The mechanisms that lead to the faster clothianidin degradation in NFA and NFC in the presence of fertiliser thus remain unknown.

Our initial hypothesis was that protection against photodegradation would occur as long as clothianidin is associated with the nanocarriers, leading to little or no protection in the low concentration range and greater protection in the high concentration range or in the presence of fertiliser. A protective effect was indeed observed at high concentration but a catalytic effect was observed in the presence of fertiliser (for NFA and NFC). There was no effect of the commercial formulation on photodegradation in any of the conditions investigated. The impact of nanoformulations on photodegradation was overall moderate, with a maximum increased of DT_50_ by 21% (NFC at the highest concentration) and maximum decrease by 24% (NFC with fertiliser) relative to the conventional formulation.

In cases where it is not possible to measure the release rate of an AI from a nanocarrier, the durability of a nanopesticide may be estimated indirectly through other kinetic phenomenon (as previously illustrated for degradation in soil^6^). The relatively small impact of the nanoformulations studied here on the photodegradation of clothianidin suggests that the release rate is likely to be fast when the formulation is diluted in an aqueous solution. Hence, the durability of the nanoformulation is expected to be relatively short (including at high concentration and high ionic strength). According to the scheme recently presented in Walker *et al.*,^2^ an environmental exposure assessment based on the pure AI or a non-nano formulation would thus likely be adequate for the nanoformulations studied here.

### Sorption

**Fig. 3:**
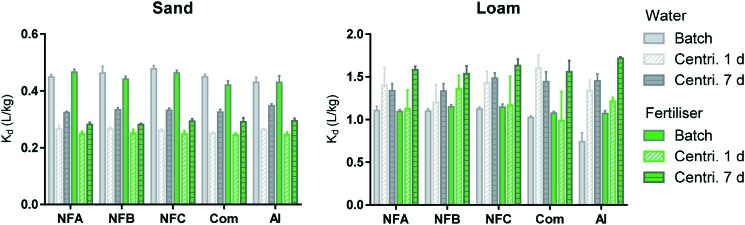
Sorption coefficients (*K*_d_, L kg^–1^) measured by batch method (plain) and centrifugation method after 1 (diagonal strips) or 7 days (horizontal strips). Data obtained in water are in grey and with fertiliser, in green. Error bars represent standard deviation for triplicates (all values are available in Table S6†).

The sorption coefficients presented in Fig. 3 (0.25 < *K*_d_ < 1.71 L kg^–1^) are characteristic of clothianidin, which is considered to be moderately mobile in the environment.^7^ Sorption in the Loam was much higher than in the Sand probably due to the higher organic carbon and clay content of the Loam. A three-way analysis of variance performed on the data obtained from the batch, and centrifugation technique after 1 and 7 days clearly shows the dominant effect of the soil type on sorption coefficients (soil accounts for more than 90% of the variation).

Comparing data obtained by centrifugation after 1 day and 7 days (bars with diagonal and horizontal strips in Fig. 3, respectively) shows that sorption significantly increased over time in the Sand, but not in the Loam (statistical comparisons are presented in Fig. S3†). An increase in sorption with time of incubation has been frequently reported previously, including when applying the centrifugation technique used in the present study.^14^,^15^,^33^ It is typically explained by the slow diffusion of the sorbate into soil aggregates, organic matrices or particle pores.^33^,^34^ Sorption kinetics are expected to be slower in the Loam than in the Sand and it is thus not known why the increase in sorption over time was only visible in the Sand. The main objective when applying the centrifugation method over time was to evaluate the possibility of estimating the release rate of the AI from the nanocarriers. There were no clear differences in the sorption kinetics of the formulations relative to the pure AI, which can be interpreted as a short durability of the nanoformulations.

The batch and centrifugation methods generated significantly different sorption coefficients (plain and patterned bars in Fig. 3, respectively). In the Sand, *K*_d_ (batch) > *K*_d_ (centrifugation), whereas in the Loam, *K*_d_ (batch) < *K*_d_ (centrifugation). In the literature, greater sorption was often measured by batch, and this was explained by an increased availability of sorption sites due to the destruction of soil aggregates during vigorous shaking.^14^,^15^,^33^,^35^ The higher *K*_d_ values measured here by centrifugation in the Loam could be explained by a rapid uptake of spiking solution into soil aggregates, the impact of suspended soil colloids (artificially decreasing *K*_d_ values measured by batch), and/or precipitation of AI due to local exceedances of clothianidin solubility limit at high soil : solution ratios.^14^,^15^,^33^ Differences between the batch and centrifugation methods were independent from the type of formulation and background solution (see Fig. S4†) and these results may thus indicate again that the durability of the nanoformulations was short, in a very diluted system (batch) as well as at realistic soil to solution ratio (centrifugation method).

A three-way analysis of variance indicated that the type of formulation had a significant effect on the sorption measured by batch and by centrifugation after 7 days. The effects that nanoformulations had on the sorption of clothianidin were however not consistent throughout the conditions investigated (all statistical comparisons are presented in Fig. S5†). In the batch, the nanoformulations had the tendency to increase sorption relative to the AI and commercial formulation (up to +51% and +10%, respectively). The only consistent trend across experimental settings was *K*_d_ (NFC) > *K*_d_ (Com). When measured by centrifugation after 7 days, sorption of the nanoformulations tended to be weaker than that of the AI (up to –7%). Larger differences between formulations were expected in the experiments conducted with the fertiliser background (due to the possible aggregation of the nanocarriers and entrapment of the AI), but this was not the case. Differences in sorption amongst formulations were not related to differences in pH (Fig. S6†). The greatest impact of formulation was observed when applying the batch method to the Loam in the water background: all formulations significantly increased the sorption of the AI (up to 51% and 38% for the nano and commercial formulation, respectively).

When considering the results reported previously for the same series of nanocarriers loaded with another AI (bifenthrin^6^), we notice that NFC tends to systematically have the greatest impact on the behaviour of both AIs. For both AIs, NFC could protect the AI from degradation (in soil^6^ and by photodegradation in the present study), but the effect on sorption depended on the AI properties. More generally, when considering results published to date on the effects of nanoformulations on sorption, it appears that decrease in sorption was observed for strongly sorbing pesticides including paraquat^36^,^37^ and bifenthrin,^6^ whereas enhanced sorption was reported for weakly sorbing AIs (*e.g.* atrazine,^14^,^38^ 2,4-D^39^) and clothianidin in the present study. Nanoformulations may thus allow the mitigation of extreme characteristics of some pesticide active ingredients, which could be valuable if the changes in the fate processes can be well controlled.

Overall, the impacts that nanoformulations had on the sorption of clothianidin were greater than that of the commercial formulation, but they remained relatively moderate. Differences among formulations were smaller when investigated by the centrifugation technique (realistic soil : solution ratio) than by the batch method (soil suspension), suggesting that the effects of formulations observed in the laboratory may be attenuated under field conditions.

## Conclusions

Our experiments on photodegradation and sorption behaviour of clothianidin suggest that nanoformulations may have a greater impact on the environmental fate of pesticide AI than commercial formulations. The type and extent of the impact are difficult to predict from the characteristics of the nanoformulation, and are most likely due to complex interactions between the formulation components (*e.g.* surfactants, polymers), the AI and the soil particles (when present). It is key to acknowledge that differences in photodegradation and sorption were relatively moderate, including when considering realistic worst-case conditions (high pesticide concentration and ionic strength). The results thus suggest that the AI clothianidin was rapidly released from the nanocarrier systems, and that the durability of the three nanoformulations would be short in water as well as in soil (including under realistic soil to solution ratio). It is essential that studies reporting only small differences between nano and non-nano counterparts are publicised in order to avoid biased interpretations of the existing state of knowledge and unfunded expectations on the possible effects that nanoformulations may have on the fate of agrochemicals.

The study illustrates how classical protocols that were initially designed for solutes can be applied as a first step to identify further requirements for regulatory assessment and decision making. Following the scheme recently proposed by a group of stakeholders for the exposure assessment of nanopesticides,^2^ a classical exposure assessment procedure would probably be adequate for the series of products presented, which is a key step towards placement on the market.

The type of nanoformulations studied here only represents an example of nanopesticides and the conclusions should not be extrapolated to other products. This study exclusively focussed on the fate of the substance with pesticidal activity and thus considered to be the most toxic. The fate of the nanocarriers is also of great interest and should be the topic of further research, not only with the objective to assess risk, but also to support a more informed development of novel nano-enabled products.

## Conflicts of interest

There are no conflicts of interest to declare.

## Supplementary Material

Supplementary information
