# Supplementary material for: Environmental fate of nanopesticides: durability, sorption and photodegradation of nanoformulated clothianidin
Source: Environ Sci Nano. 2018 Feb 22;5(4):882–9. doi: 10.1039/c8en00038g (PMC5918303; doi:10.1039/c8en00038g)
Supplement: Supplementary file 1 [file EN-005-C8EN00038G-s001.pdf]

Electronic Supplementary Material (ESI) for Environmental Science: Nano.
This journal is © The Royal Society of Chemistry 2018

Supporting Information

Environmental fate of nanopesticides: durability, sorption and photodegradation of nanoformulated clothianidin

Melanie Kah[a,b], Helene Walch[a], Thilo Hofmann[a]

[a]University of Vienna, Department of Environmental Geosciences and Environmental Science Research Network, Althanstrasse 14, 5 UZA2, 1090 Vienna, Austria

[b]Commonwealth Scientific and Industrial Research Organisation (CSIRO), Waite Campus, Locked Bag No 2, Glen Osmond, SA 5064, Australia

melanie.kah@univie.ac.at

Table of Contents

Table S1 Soil properties (± standard deviation) S2
Table S2 Composition of the formulations.. .... S2
Table S3 Hydrodynamic diameter, polydispersity index and ζ-potential measurements S3
Table S4 Foliar spray (photodegradation) and in-furrow seed treatment (sorption) S4
Table S5. Photodegradation half-lives (DT_{50} in days ± standard deviation) S5
Table S6. Sorption coefficient (K_d values, L/kg ± standard deviation) S5

Figure S1. Examples of photodegradation curves (n=3) fitted with first order kinetics. S6
Figure S2. Comparisons of photodegradation rate constants for the different formulation S7
Figure S3. Comparisons of sorption over time as measured by centrifugation after 1 and 7 days S8
Figure S4. Comparisons of methods: batch vs centrifugation 7 days S9
Figure S5. Comparisons of the formulations across a range of conditions S10
Figure S6. pH values of the soil suspensions when measuring sorption by batch. S11

References S12

Table S1 Soil properties (± standard deviation)

Soil type[a]	%OC[b]	N	pH[c]	CEC (meq/100g]	% Clay[a] (<2 µm)	% Sand[a] (0.05-2 mm)	MWHC[d] (g/100g)
Sand	0.65±0.1	0.05±0.01	5.1±0.3	4.3±0.5	2.8±1.1	87.0±1.5	31.1±2.1
Loam	2.26±0.5	0.20±0.04	7.2±0.2	31.4±4.6	25.9±2.1	33.6±1.8	44.1±1.2

[a] according to USDA
[b] difference in carbon content (heat-conductivity detector) before and after combustion of the soil samples at 425°C
[c] determined at a soil:solution mass-ratio of 10:25 in 0.01 M $CaCl_2$
[d] Maximum Water Holding Capacity: amount of water a soil sample can hold against gravity when left to drain for 2h on a saturated sand bath (ISO 11268-2 2012)

Table S2 Composition of the formulations. + indicates the presence and - the absence of a component.

Ingredients	NFA	NFB	NFC
Water	+	+	+
Acrylates polymer	+	+	+
Sodium methyl oleoyl taurate	-	+	-
Sodium alkylnaphthalenesulfonate, formaldehyde condensate	+	-	+
Propylene glycol	-	-	-
Clothianidin content (% weight) as analysed by HPLC	19.19	20.50	19.11

The commercial formulation Belay contained 23.60% of clothianidin (% weight) as measured by HPLC. The designation “suspension concentrate” describes a stable suspension of active ingredient(s) in an aqueous continuous phase and is based on parameters related to pourability and water dispersibility[1]. The specific characteristics of suspension concentrates are typically achieved by addition of dispersant(s) and wetting agent(s) with polymeric viscosity stabiliser(s)[2]. The exact ingredients contained in Belay are unknown (proprietary blend).

Table S3 Hydrodynamic diameter, polydispersity index and ζ-potential measurements

Methods: Measurements were carried out in 1:400 (volume based) dilutions by mixing a stock solution of each formulation (1:25) with a particular background solution directly in the measurement cell. The final NaCl and $Ca(NO_3)_2$ concentrations were 190 and 75 mM, respectively. ζ-potentials were also measured at 0.1 mM NaCl. Regarding fertilizer background, measurements could not be conducted at the exact experimental conditions due to the low concentrations of nanopesticide. Hence, measurements were conducted at a dilution of 1:400 as above (about 3.1, 8.4 and 76.9 fold more concentrated than in the photodegradation, centrifugation and batch sorption tests, respectively) but maintaining the experimental fertilizer-to-formulation ratios.

The hydrodynamic diameter was measured immediately upon mixing (crosses on Figure 1), and included 10 individual measurements to resolve aggregation (3 seconds acquisition time for each individual run), followed by 3-6 individual measurements (10 seconds acquisition time for each individual run). The last three runs were stacked and averaged to determine the final size (> 8 min after mixing, bars in Figure 1). After size measurements, sample aliquots were transferred into folded capillary cells (Malvern DTS1070) for ζ-potential measurements in triplicates.

**Hydrodynamic diameter and polydispersity width[a] (nm ± standard deviation, n=3, after >8 min)**						
		Deionised water	190 mM NaCl	75 mM $Ca(NO_3)_2$	Photodegradation	Sorption
NFA	H. diam.	1264.3±31.6	1228.7±19.0	874.8±14.2	3039.3±224.7	1090.3±32.5
	Pd width	270.6±164.8	492.9±76.1	155.4±75.5	1997.7±1036.1	309.9±112.9
NFB	H. diam.	929.5±41.1	953.7±35.2	1177.0±127.6	2768.2±427.7	1323.0±75.1
	Pd width	342.5±56.7	194.0±99.9	332.1±223.4	1212.7±483.7	516.4±134.7
NFC	H. diam.	899.9±46.3	860.8±13.6	2648.0±313.0	3167.0±282.8	1529.3±37.0
	Pd width	308.2±29.7	314.9±73.4	1759.3±776.5	1122.8±366.1	368.9±229.3
Com	H. diam.	882.3±36.1	1464.0±10.0	1469.3±182.4	3175.2±1020.0	1640.3±26.8
	Pd width	320.8±61.7	552.1±99.8	849.9±183.5	2434.1±1269.6	491.2±112.8

[a] Polydispersity width: square-root of the polydispersity index times z-average

**ζ-potential (mV ± SD, n=3)**						
	Deionised water	0.1 mM NaCl	190 mM NaCl	75 mM $Ca(NO_3)_2$	Photodegradation	Sorption
NFA	-59.7±0.4	-14.9±0.3	-14.4±0.9	-2.7±0.3	-10.1±0.2	-3.1±0.2
NFB	-62.3±1.9	-61.9±1.2	-28.9±0.7	-10.4±1.3	-13.0±1.6	-21.0±1.0
NFC	-64.6±0.6	-63.4±0.6	-32.5±1.5	-9.6±0.5	-18.5±3.1	-36.7±3.6
Com	-28.6±0.3	-27.3±0.6	-4.9±0.9	-1.9±0.1	-6.1±2.1	-5.4±1.4

	**Electric Conductivity [µS/cm]**		**pH[a]**
	0.1 mM NaCl	Deionised water	Deionised water
NFA	148.2	132.8	4.33
NFB	96.1	81.2	5.36
NFC	90.0	77.0	7.61
Com	19.0	4.4	6.07

[a] Measured at the1:400 (volume based) dilution. Note that pH tended to increase with increasing concentration for NFC and Com, whereas it decreased for NFA, and remained stable for NFB.

Table S4 Concentrations of clothianidin and fertiliser considering foliar spray (photodegradation) and in-furrow seed treatment (sorption)

	**Photodegradation**<br>Foliar spray scenario<br>Conc. in the spraying tank (mg/L)	**Sorption**<br>In-furrow scenario<br>Conc. in soil (mg/kg)
Clothianidin	136	13
NH_4^+	18400	320
P_2O_5	62560	1088
Fe^{2+}	184	3.2

Photodegradation: foliar spray application scenario

Clothianidin is most likely to be exposed to sunlight when applied as foliar spray, possibly together with some fertilisers. Concentrations of nutrients were calculated based on the maximum recommended application rate as foliar spray 40 L of fertiliser/ha (applied before full coverage on corn and sugar beet) diluted in a minimum of 300 L water/ha[3]. According to the label of the insecticide Belay (clothianidin content: 2.13 lbs/gal = 255.23 g/L) the maximum application rate for most crops is 6 fl.oz./A, diluted in a minimum of 100 gal of water/A, which gives a spray concentration of 120 mg/L. Note that for crops such as grapes and pome fruits, concentrations could be even higher (200-480 mg/L)

Sorption to soil: in-furrow application scenario

The high concentration of fertilizer and clothianidin in soil are most likely to occur when applied in-furrow while sowing. Experimental concentrations were based on the maximum realistic usage rate. When the fertilizer PowerPhos is used for basic soil fertilization, the label recommends using 40 L/ha e.g. in corn, potatoes, sunflowers, oil-pumpkins and sugar beets[3]. Clothianidin is often used for seed treatment of corn and the scenario was thus developed for corn. Applying 40 L/ha in-furrow would lead to very high local salt concentrations, possibly causing salt injury of the seeds. However, the recommended application rate for 10-34-0 fertilizer in-furrow on corn is 5 gal/A (≈ 47 L/ha) in 30-inch (≈ 76 cm) rows [4], indicating that the scenario is realistic.

With an average number of 10 plants per m^2 and 75 cm row distance, planting distance within rows needs to be about 13 cm [5]. Each plant accounts for 13 cm of furrow, so the total furrow length is 10 plants × 13 cm = 130 cm/m^2. It was assumed that 3 × 3 cm of soil is influenced along the furrow length. The volume of influenced soil would then be 11.7 m^3/ha. The fertiliser should be mixed with a minimum of 300 L water per ha[3], giving a total of 340 L/ha. With a concentration of 138 g of N/L , the scenario results in 315 mg of N/kg in dry soil, assuming a soil bulk density of 1.5 kg/dm^3. We used 320 mg of N/kg N and corresponding 1088 mg of P2O4/kg (NPK ratio: 10-34-0).

Similar considerations were made to calculate the concentration of clothianidin in soil: in-furrow application of Belay against corn wireworm is 12 fl.oz./A (= 0.8769 L/ha). The insecticide contains 2.13 lbs clothianidin per gallon (= 255.23 g/L)[6], so the maximum application amounts to 223.81 g/ha. Considering the influenced volume of soil 11.7 m^3/ha (as calculated above), clothianidin concentration in soil would be 19.13 g/m^3, equivalent to ~13 mg/kg considering a soil density of 1.5 g/cm^3. Experimental concentrations of clothianidin and fertilizer in soil are summarised in the Table above.

Table S5. Photodegradation half-lives (DT_{50} in days ± standard deviation) for the series of nanoformulations (NFA, NFB and NFC), the commercial formulation (Com) and the pure AI (data presented in Figure 2 in the manuscript)

Clothianidin (mg/L)	Background	**DT_{50} (h)**				
		**NFA**	**NFB**	**NFC**	**Com**	**AI**
13.6	Water	1.00 ± 0.05	0.97 ± 0.06	1.06 ± 0.07	0.96 ± 0.06	0.92 ± 0.05
102		1.28 ± 0.06	1.25 ± 0.07	1.28 ± 0.10	1.25 ± 0.07	1.17 ± 0.07
136		1.49 ± 0.10	1.44 ± 0.09	1.47 ± 0.10	1.30 ± 0.08	1.33 ± 0.08
203				1.68 ± 0.11	1.39 ± 0.07	1.35 ± 0.10
306				2.06 ± 0.11	1.77 ± 0.09	
408		2.49 ± 0.17	2.31 ± 0.08	2.46 ± 0.11	2.15 ± 0.07	
544		3.47 ± 0.26	3.43 ± 0.15	3.61 ± 0.14	2.99 ± 0.13	
136	Fertiliser	5.61 ± 0.29	6.49 ± 0.19	4.72 ± 0.23	6.25 ± 0.14	6.44 ± 0.21

Table S6. Sorption coefficient (K_d values, L/kg ± standard deviation) measured for the three nanoformulations (NFA, NFB, NFC), a commercial formulation (Com) and pure clothianidin (AI). Measurements were performed in two soils by batch and by centrifugation technique after 1 and 7 days.

		**K_d (L/kg)**					
		**NFA**	**NFB**	**NFC**	**Com**	**AI**	**Average**
Batch	loam fertiliser	1.094 ± 0.021	1.147 ± 0.027	1.146 ± 0.034	1.075 ± 0.021	1.071 ± 0.033	1.062
	loam water	1.103 ± 0.051	1.099 ± 0.026	1.121 ± 0.024	1.022 ± 0.020	0.741 ± 0.103	
	sand fertiliser	0.466 ± 0.010	0.441 ± 0.010	0.463 ± 0.010	0.420 ± 0.015	0.430 ± 0.024	0.449
	sand water	0.449 ± 0.009	0.462 ± 0.024	0.478 ± 0.012	0.449 ± 0.009	0.430 ± 0.017	
Centrifugation	loam fertiliser 1d	1.125 ± 0.219	1.359 ± 0.160	1.167 ± 0.337	0.990 ± 0.338	1.214 ± 0.045	1.283
	loam water 1d	1.400 ± 0.208	1.202 ± 0.209	1.431 ± 0.138	1.604 ± 0.151	1.339 ± 0.123	
	loam fertiliser 7d	1.583 ± 0.039	1.535 ± 0.093	1.632 ± 0.076	1.559 ± 0.133	1.717 ± 0.017	1.508
	loam water 7d	1.337 ± 0.083	1.333 ± 0.089	1.484 ± 0.064	1.444 ± 0.114	1.452 ± 0.083	
	sand fertiliser 1d	0.249 ± 0.008	0.251 ± 0.013	0.249 ± 0.011	0.245 ± 0.006	0.246 ± 0.009	0.255
	sand water 1d	0.267 ± 0.011	0.266 ± 0.007	0.260 ± 0.005	0.251 ± 0.004	0.264 ± 0.003	
	sand fertiliser 7d	0.282 ± 0.007	0.281 ± 0.004	0.293 ± 0.008	0.291 ± 0.014	0.295 ± 0.009	0.310
	sand water 7d	0.324 ± 0.004	0.334 ± 0.008	0.332 ± 0.008	0.325 ± 0.010	0.347 ± 0.007	

Figure S1. Examples of photodegradation curves (n=3) fitted with first order kinetics. The DT_{50} values derived are shown in Figure 2.

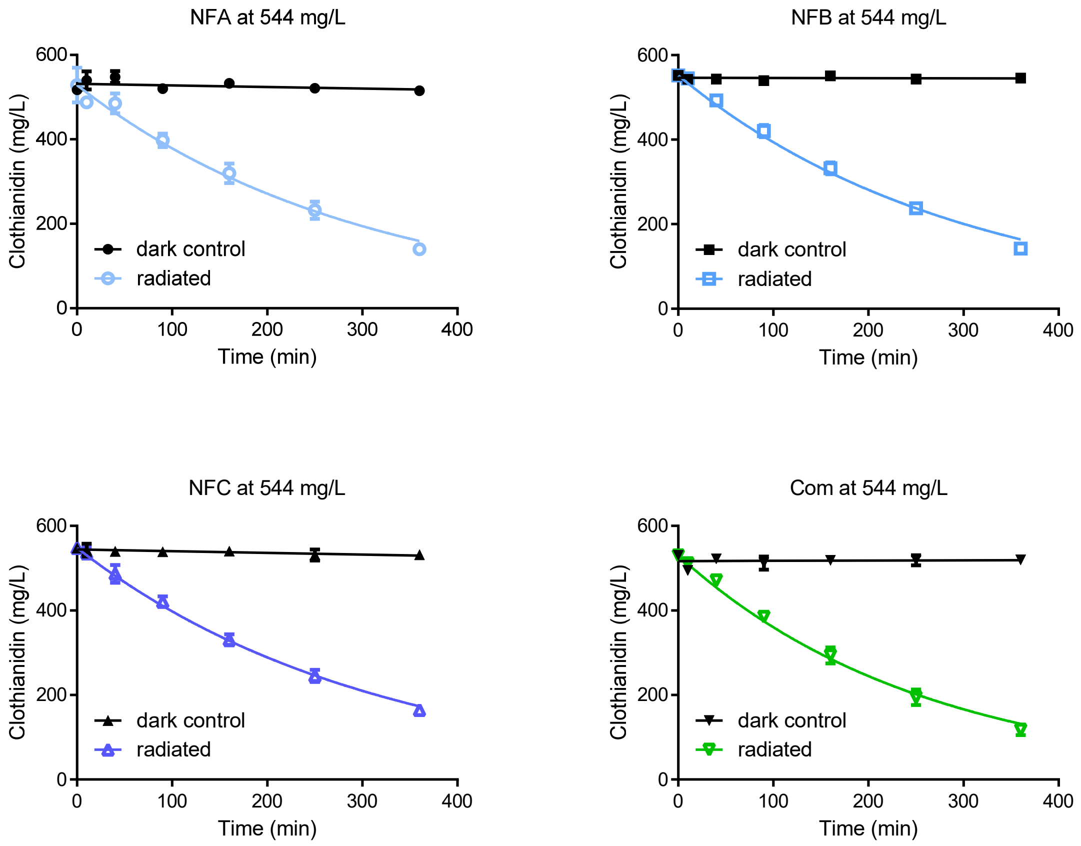

Figure S2. Comparisons of photodegradation rate constants (k, min^{-1}) for the different formulations across concentration and background (corresponding colour-coded data are presented in Figure 2)

Groups of statistically equal values are denoted by letters (statistically non-different values share at least one letter).

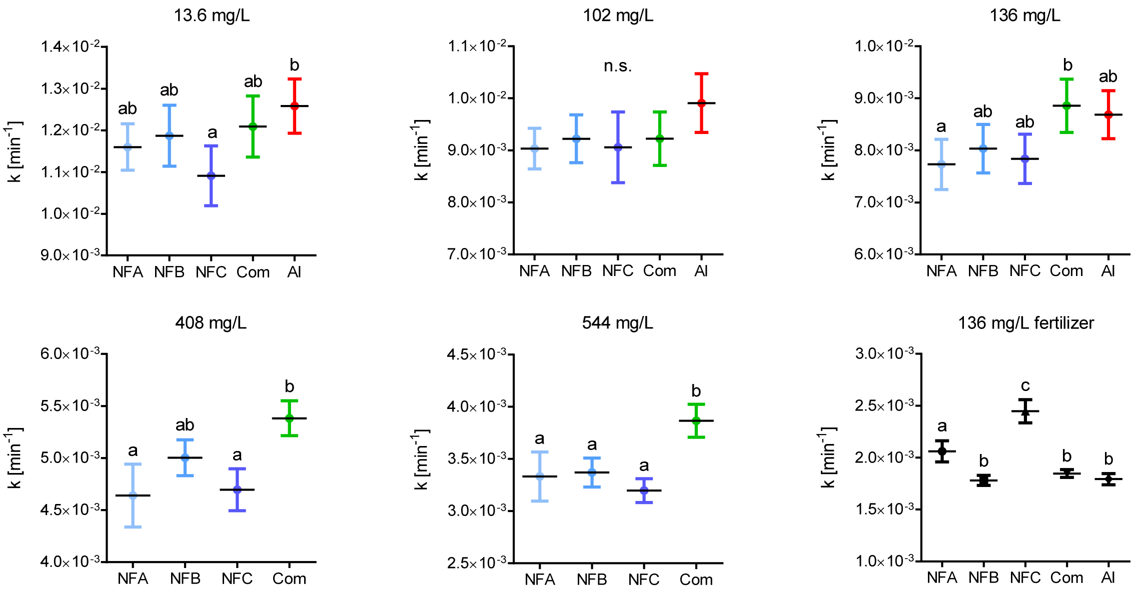

Figure S3. Comparisons of sorption over time as measured by centrifugation after 1 day (diagonal) and after 7 days (horizontal strips).
The significance level was set to α=0.05 and significant differences by * .

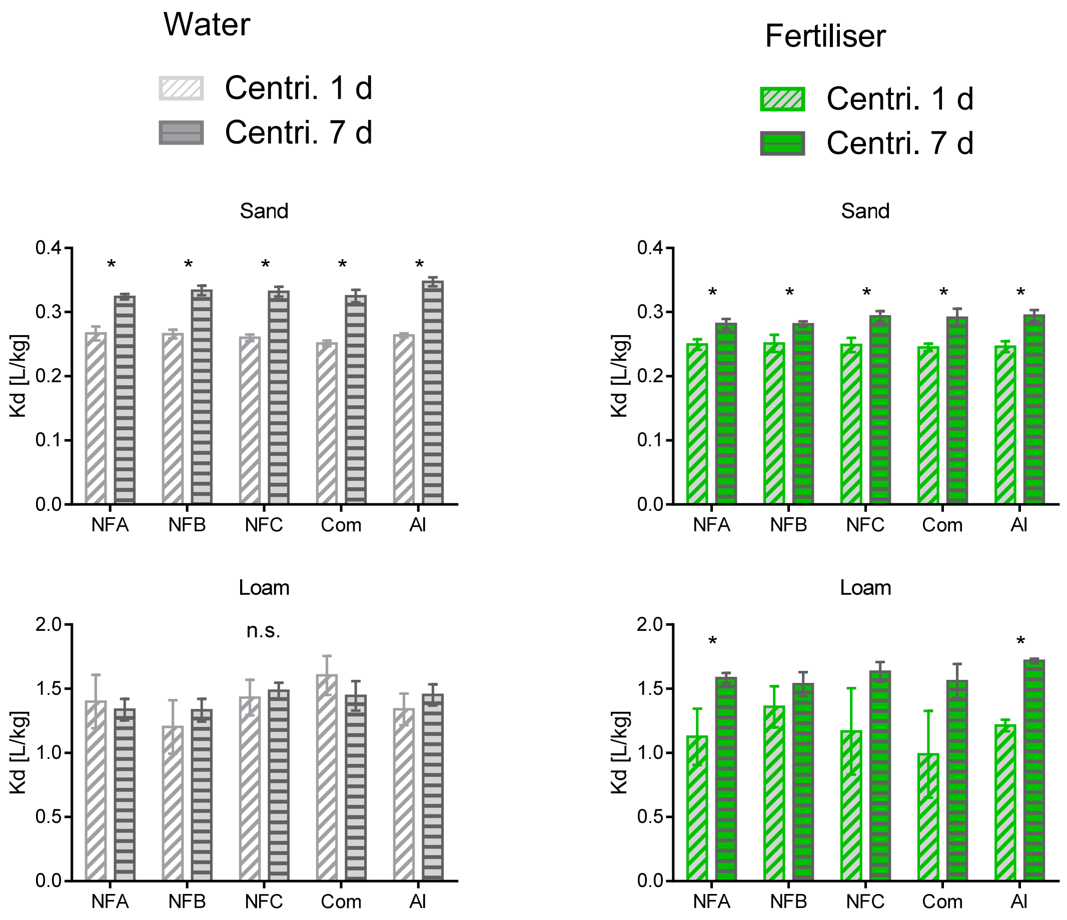

Figure S4. Comparisons of methods: batch vs centrifugation 7 days
The significance level was set to α=0.05 and significant differences by * .

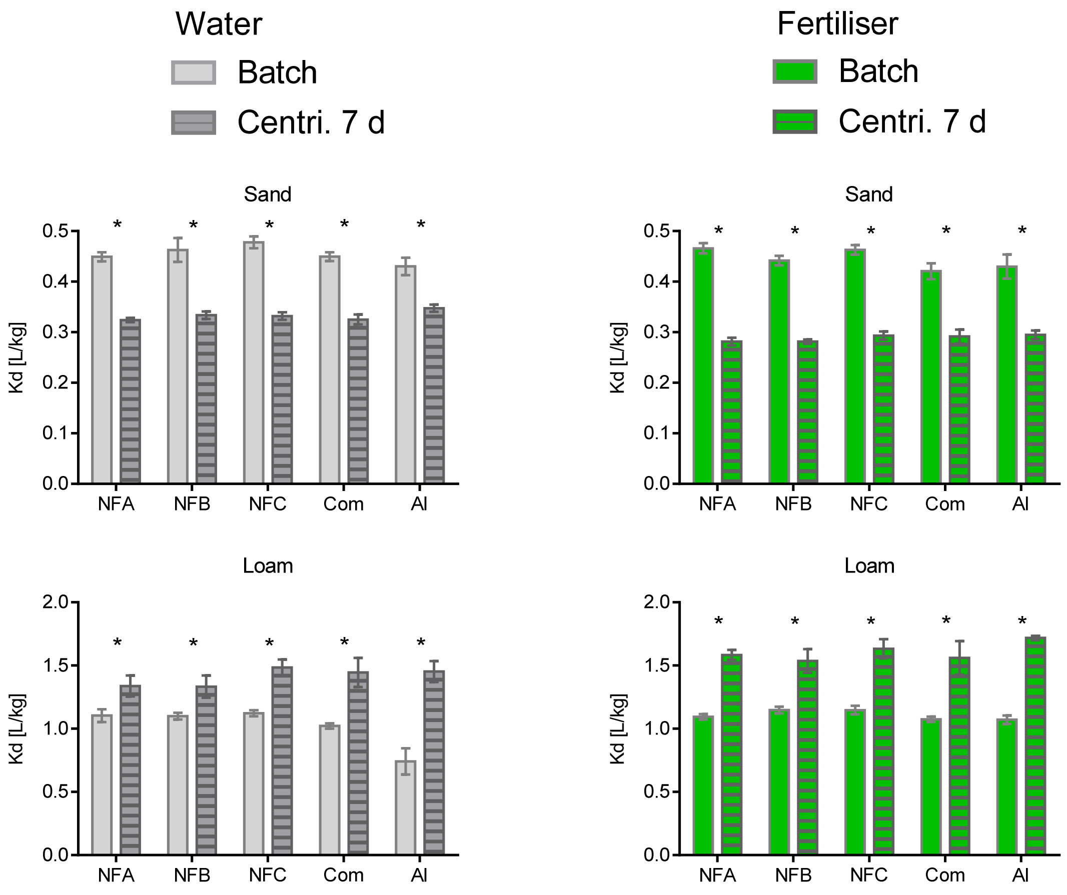

Figure S5. Comparisons of the formulations across a range of conditions
Groups of statistically equal values are denoted by letters (statistically non-different values share at least one letter).

Batch method

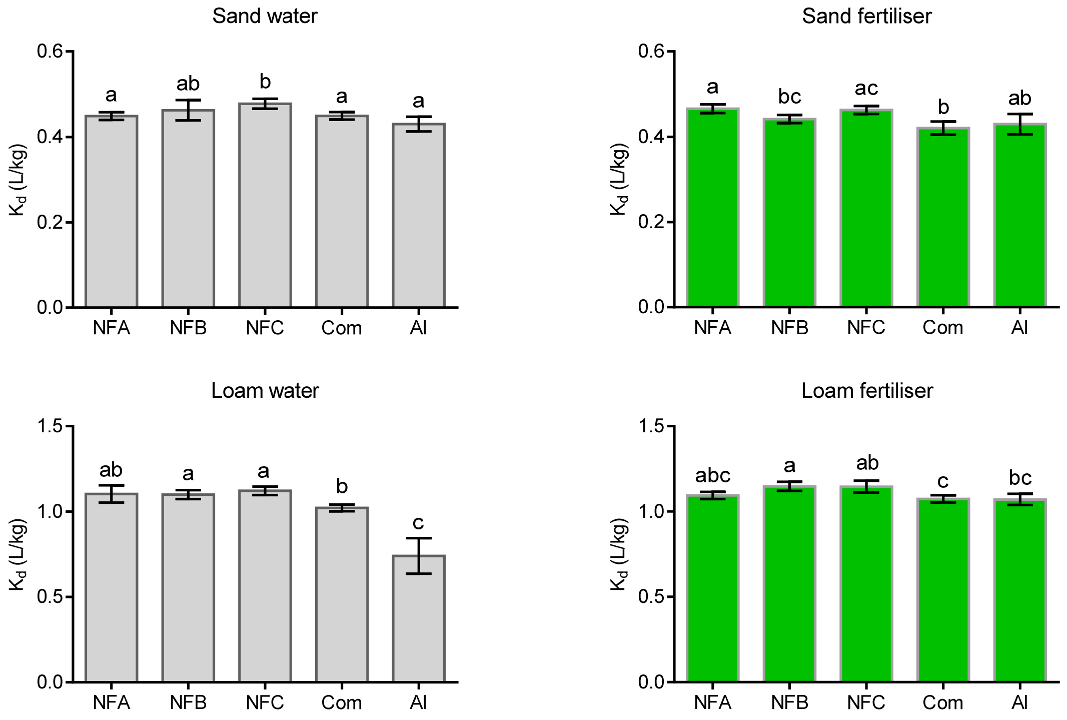

Centrifugation method (7 days)

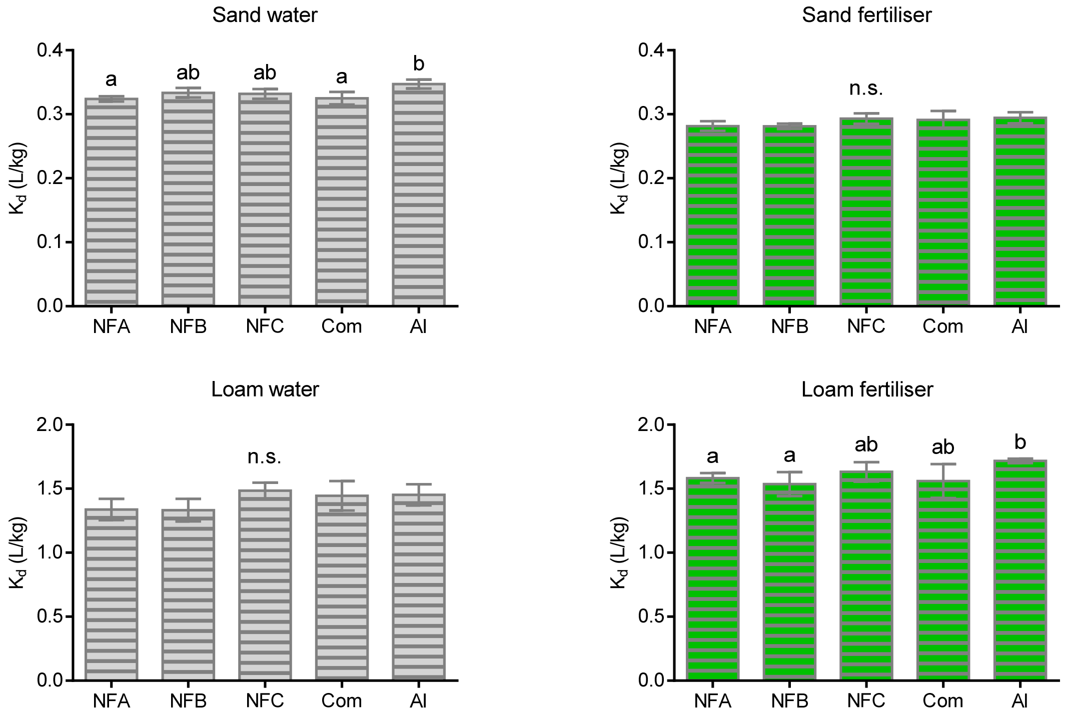

Figure S6. pH values measured after equilibration of the soil suspensions when measuring sorption by batch.

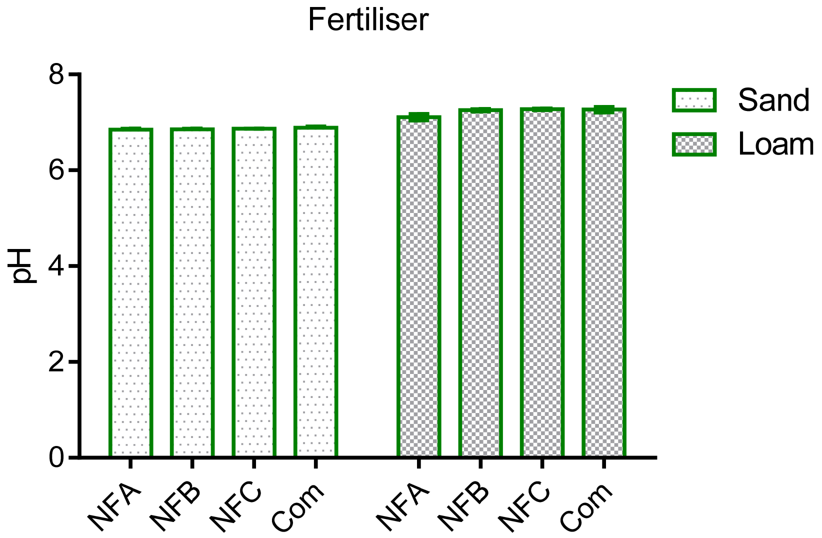

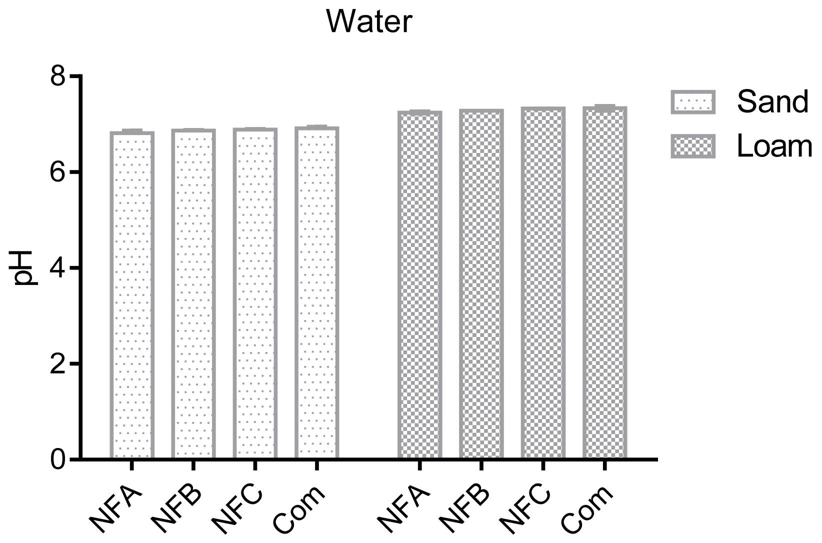

A two-way ANNOVA indicates that the effect of formulation on pH was overall not significant.

References

1 FAO/WHO, Manual on development and use of FAO and WHO Specifications for Pesticides, http://www.fao.org/fileadmin/templates/agphome/documents/Pests_Pesticides/Specs/JMPS_Manual_2016/3rd_Amendment_JMPS_Manual.pdf, (accessed February 7, 2018).

2 Gouge, T., Understanding Pesticide formulations, http://www.cdpr.ca.gov/docs/emon/surfwtr/presentations/gouge_formulation_050510.pdf, (accessed February 7, 2018).

3 Hechenbichler GmbH, Label: POWERPHOS liquid fertilizer., http://www.kroni.ch/pdfs/K%20938%20Powerphos%20Internet.pdf;, (accessed November 29, 2017).

4 G. W. Hergert, C. H. Wortmann, R. B. Ferguson, C. A. Shapiro and T. M. Shaver, Using starter Fertilizers for corn, grain sorghum, and soybeans, http://extensionpublications.unl.edu/assets/pdf/g361.pdf, (accessed August 18, 2017).

5 LWK Nordrhein-Westfalen, Bestandesdichte und Reihenabstände, https://www.landwirtschaftskammer.de/landwirtschaft/ackerbau/mais/bestandsdichte-pdf.pdf, (accessed November 29, 2017).

6 Valent USA Corp., Label: Belay Insecticide, https://www.valent.com/Data/Labels/2016-BEL-0001-R1-1681-H.pdfalent USA Corp., (accessed November 29, 2017).
